# *Saccharomyces cerevisiae* strain comparison in glucose–xylose fermentations on defined substrates and in high-gravity SSCF: convergence in strain performance despite differences in genetic and evolutionary engineering history

**DOI:** 10.1186/s13068-017-0887-9

**Published:** 2017-09-04

**Authors:** Vera Novy, Ruifei Wang, Johan O. Westman, Carl Johan Franzén, Bernd Nidetzky

**Affiliations:** 1 Institute of Biotechnology and Biochemical Engineering, Graz University of Technology, Graz, Austria; 2 Division of Industrial Biotechnology, Department of Biology and Biological Engineering, Chalmers University of Technology, Gothenburg, Sweden

## Abstract

**Background:**

The most advanced strains of xylose-fermenting *Saccharomyces cerevisiae* still utilize xylose far less efficiently than glucose, despite the extensive metabolic and evolutionary engineering applied in their development. Systematic comparison of strains across literature is difficult due to widely varying conditions used for determining key physiological parameters. Here, we evaluate an industrial and a laboratory *S. cerevisiae* strain, which has the assimilation of xylose via xylitol in common, but differ fundamentally in the history of their adaptive laboratory evolution development, and in the cofactor specificity of the xylose reductase (XR) and xylitol dehydrogenase (XDH).

**Results:**

In xylose and mixed glucose–xylose shaken bottle fermentations, with and without addition of inhibitor-rich wheat straw hydrolyzate, the specific xylose uptake rate of KE6-12.A (0.27–1.08 g g_CDW_^−1^ h^−1^) was 1.1 to twofold higher than that of IBB10B05 (0.10–0.82 g g_CDW_^−1^ h^−1^). KE6-12.A further showed a 1.1 to ninefold higher glycerol yield (0.08–0.15 g g^−1^) than IBB10B05 (0.01–0.09 g g^−1^). However, the ethanol yield (0.30–0.40 g g^−1^), xylitol yield (0.08–0.26 g g^−1^), and maximum specific growth rate (0.04–0.27 h^−1^) were in close range for both strains. The robustness of flocculating variants of KE6-12.A (KE-Flow) and IBB10B05 (B-Flow) was analyzed in high-gravity simultaneous saccharification and co-fermentation. As in shaken bottles, KE-Flow showed faster xylose conversion and higher glycerol formation than B-Flow, but final ethanol titres (61 g L^−1^) and cell viability were again comparable for both strains.

**Conclusions:**

Individual specific traits, elicited by the engineering strategy, can affect global physiological parameters of *S. cerevisiae* in different and, sometimes, unpredictable ways. The industrial strain background and prolonged evolution history in KE6-12.A improved the specific xylose uptake rate more substantially than the superior XR, XDH, and xylulokinase activities were able to elicit in IBB10B05. Use of an engineered XR/XDH pathway in IBB10B05 resulted in a lower glycerol rather than a lower xylitol yield. However, the strain development programs were remarkably convergent in terms of the achieved overall strain performance. This highlights the importance of comparative strain evaluation to advance the engineering strategies for next-generation *S. cerevisiae* strain development.

**Electronic supplementary material:**

The online version of this article (doi:10.1186/s13068-017-0887-9) contains supplementary material, which is available to authorized users.

## Background

Bioethanol, produced from lignocellulosic feedstock, is one of the most promising fossil fuel substitutes and it can help to mitigate climate change and secure energy supply chains [1, 2]. However, there are still major obstacles in the bioethanol production process, which have to be overcome to realize the full potential for commercialization [1, 3].

A main challenge is to find, or engineer, a fermentation organism that performs well in the difficult substrate presented by the lignocellulosic hydrolyzates [4, 5]. During the pretreatment step, high levels of inhibitory compounds (e.g., aromatic aldehydes and organic acids) are formed by secondary decomposition processes [6, 7]. The lignocellulosic hydrolyzates further contain significant concentrations of hemicellulose-derived pentoses, mainly xylose, besides the cellulose-derived glucose [3]. Realization of the full potential of the feedstock requires conversion of all provided sugars [8].

To target this, extensive research effort has been spent on enabling *Saccharomyces cerevisiae* to ferment xylose [9–11]. Based on its inherent robustness and process stability, this yeast is the preferred organism of the industries and a promising candidate for lignocellulose-to-ethanol processes [9–11]. *S. cerevisiae*, however, is naturally unable to ferment xylose [12, 13], necessitating the introduction of a heterologous xylose assimilation pathway into the yeast’s genome. Two different pathways are available; the bacterial direct isomerization of xylose-to-xylulose, catalyzed by xylose isomerase (XI) [14], and the fungal “net” isomerization in two oxidoreductive steps via xylitol, catalyzed by xylose reductase (XR) and xylitol dehydrogenase (XDH) [9–11]. Both strategies have resulted in strains with the desired xylose-converting phenotype [9–11, 14]. Despite the recent success of strains harboring the XI [15–17], the XR/XDH pathway remains a strong option for development [9–11, 18].

Irrespective of the basic engineering strategy applied, however, the resultant strains display specific xylose uptake rates (*q*_Xylose_) considerably lower than the corresponding glucose uptake rates [9–11]. A substrate uptake rate is a complex manifestation of the microbial physiology and may be limited by the actual uptake into the cell, metabolic integration, or both. It is often more convenient to try to evolve a complex physiological parameter rather than engineer it rationally [19]. Strategies applied to improve *q*_Xylose_ in *S. cerevisiae* include evolution in repetitive batch cultivations [20–22], continuous chemostat experiments [23, 24], or a combination of the two [25]. Strain selection has mainly been based on aerobic [21, 22] or anaerobic growth on xylose [20, 23]. Laboratory evolution has been further applied to increase the yeast’s tolerance against the stressors and inhibitors present in the lignocellulosic substrates [24–26].

The main difficulty of evolutionary engineering lies in the proper choice of both selection pressure and screening parameter [19, 27]. According to the slogan “you only get what you screen for,” strains evolved for improved aerobic growth on xylose, might not actually show an improved anaerobic specific rate of ethanol production (*q*_Ethanol_), and an accelerated *q*_Xylose_ might result in decreased ethanol yields (*Y*_Ethanol_) [27]. Furthermore, strains are often characterized only under a few cultivation conditions [10, 27]. Because the maximum specific growth rate (*µ*_max_), *q*_Xylose_, and *Y*_Ethanol_ are highly dependent on the experimental set-up (e.g., sugar substrate concentrations, pH, inhibitor content, cell density), broad variation in the experimental conditions across literature makes a rigorous comparison of the different strains difficult.

Another challenge in advancing large-scale bioethanol production from lignocellulosic feedstock is to achieve the high final ethanol titers necessary to render the process cost-effective (40–50 g L^−1^, e.g., [8]). This requires the processing of high solid loadings which is associated with problems such as high concentrations of inhibitors [6, 28], mass and heat transfer limitations due to high viscosities [29], and insufficient xylose fermentation caused by high glucose-to-xylose ratios [30, 31]. Fed-batch simultaneous saccharification and co-fermentation (SSCF), with substrate, enzyme, and cell feeding, or a combination thereof, has been shown to be useful to overcome these problems [24, 28–31].

In this study, we compare two xylose-fermenting strains of *S. cerevisiae*, IBB10B05 [20] and KE6-12.A ([25], Albers et al., unpublished), that were established independently through completely different development programs. Both strains harbor the XR/XDH pathway and were evolved for growth on xylose and accelerated xylose conversion ([20, 25], Albers et al., unpublished) but they differ fundamentally in their metabolic and evolutionary engineering history. Strain characterization was conducted in anaerobic shaken bottle experiments on synthetic sugar substrates with and without addition of inhibitor-rich wheat straw hydrolyzate. This allowed for precise determination of the metabolite yields, the growth rates, and the specific substrate uptake rates. To further compare the strains in a process set-up closer to industrial applications, the severity of the fermentation conditions was increased and flocculating variants of IBB10B05 (B-Flow) and KE6-12.A (KE-Flow) were applied in high-gravity multi-feed SSCFs. This study will give insights into how the specific traits of the two strains, which were elicited by different metabolic and evolutionary engineering strategies, can affect the global fermentation performance under laboratory conditions and in industrially relevant experimental set-ups.

## Methods

### Strains

The genetically and evolutionary engineered *S. cerevisiae* strains IBB10B05 (Graz University of Technology, Austria) and KE6-12.A (Chalmers University of Technology, Sweden) were used. IBB10B05 is a descendant of BP10001, which was enabled to xylose fermentation by the genomic integration of a mutated (K274R; N276D) XR variant from *Candida tenuis*, the wild-type XDH from *Galactocandida mastotermitis* and an additional copy of the endogenous xylulose kinase 1 [32]. Evolutionary engineering of BP10001 was described before [20], and will be only briefly summarized in the following. Throughout the evolution procedure, mineral medium was utilized with xylose as sole carbon source (XM). The pH was stabilized at 6.5 with K_2_HPO_4_ buffer and incubation was under strictly anaerobic conditions at 30 °C. BP10001 was firstly cultivated in a batch culture for 91 days. Subsequently, cells were transferred to XM-agar plates. The fastest growing colony was subjected to further engineering by repetitive batches. After several rounds, the clone showing the highest μ_max_ and *q*_Xylose_ was IBB10B05. In total IBB10B05 was evolved from BP10001 in 61 generations [20].

KE6-12.A is a non-commercial strain derived from TMB3400 by evolutionary engineering [25]. TMB3400 was generated by genomic integration of *Pichia stipitis* XR and XDH genes, and a combination of chemical mutagenesis and laboratory evolution was then used [21]. TMB3400 was further evolved resulting in KE6-12.A, and a detailed description of the secondary evolution procedure will be published elsewhere (Albers et al., unpublished). In short, the parent strain (obtained after initial evolutions with heat treatment and high xylose levels for 15 and 77 generations) was cultivated in a continuous culture at pH 5.0 and 35 °C. The cultivation was started with a batch phase without any air inflow using glucose and xylose-based mineral medium. Subsequently, the continuous phase was initiated by feeding xylose with increasing levels of inhibitor-rich bagasse hydrolyzate. The cultivation was run as a turbidostat with low aeration. During the continuous phase, the last strain saved as frozen stock contained a mixed population (denoted KE6-12), generated after 120 generations. In a later study, the best performing single cell line was singled out and denoted KE6-12.A [25].

In SSCF experiments, flocculating variants of IBB10B05 and KE6-12.A were used. The strains were made flocculating by genomic integration of the *FLOw* gene at the *HO* locus [33]. The resulting flocculating IBB10B05 and KE6-12.A were denoted B-Flow and KE-Flow, respectively.

### Raw materials

The liquid and the solid fractions of pretreated wheat straw were obtained from SEKAB E-technology (Örnsköldsvik, Sweden). The wheat straw was pretreated by acid-catalyzed (0.2% (w/v) H_2_SO_4_) steam explosion. After pretreatment, the slurry was separated by press filtration into a xylose- and inhibitor-rich liquid (denoted herein hydrolyzate) and a cellulose-rich solid fraction. The two fractions were used independently in this study. The pretreatment strategy will be published in detail in a separate publication [33]. The compositions of both fractions are summarized in Additional file 1: Table S1. Prior to use, the pH of the liquid fraction was adjusted to 6.5 with NaOH, after which it was sterilized using 0.45 µm filters (Klari-Flex, Whatman, Maidstone, United Kingdom).

### Shaken bottle fermentations

#### Media

Unless otherwise stated, all chemicals were from Carl Roth + Co KG (Karlsruhe, Germany). YPD medium contained 10 g L^−1^ yeast extract, 20 g L^−1^ casein peptone, and 20 g L^−1^ glucose. YPD agar plates additionally contained 20 g L^−1^ agar. YX, YG, and YGX media contained yeast extract (10 g L^−1^) and the carbon sources xylose (40 g L^−1^), glucose (40 g L^−1^), and a combination thereof (40 g L^−1^ xylose, 40 g L^−1^ glucose), respectively. Fermentations conducted in a hydrolyzate matrix contained 70 vol% hydrolyzate (H), 10 vol% yeast extract solution (10 g L^−1^), 10 vol% sugar solution, and 10 vol% inoculum. Xylose was added to the H-YX medium to reach a final concentration of 30 g L^−1^. Glucose and xylose were added to the H-YGX medium to reach final concentrations of 40 and 30 g L^−1^, respectively. Because of the low concentration of glucose in the hydrolyzate (Additional file 1: Table S1), H-YX media additionally contained ~2 g L^−1^ glucose. Low cell density fermentations were additionally supplemented with 0.1 vol% ergosterol solution (10 g L^−1^ ergosterol, 420 g L^−1^ Tween-80, both Sigma-Aldrich, St. Louis, MO, USA, boiled in 96 vol% ethanol).

#### Fermentations

Cells were stored in glycerol stocks and initially plated on YPD agar plates. Incubation was at 30 °C for 48 h. Cells were then used to inoculate 50 mL of YPD medium in 300 mL baffled shake flasks. Incubation was at 30 °C overnight. Cells were transferred to 300 mL of YPD medium in 1000 mL baffled shake flasks to a starting OD_600_ of 0.05, and incubated at 30 °C. Cells were harvested within the exponential growth phase (OD_600_ < 2.5) by centrifugation (4420*g*, 4 °C, 20 min, Sorvall RC-5B) and the cell pellet was washed and resuspended in 0.9% (w/v) NaCl solution. Reactions were performed anaerobically at 30 °C in glass bottles, tightly sealed with rubber septa (90 mL working volume). The bottles were sparged with N_2_ prior to and shortly after inoculation. Starting OD_600_ was either 5 (high cell density fermentations) or 0.1 (low cell density fermentations). Incubation was performed at 180 rpm in a CERTOMAT BS-1 incubator shaker (Sartorius AG, Göttingen, Germany).

#### Analysis of cell growth, cell viability, sugars, and metabolites

Samples of 1.5 mL were frequently removed from shaken bottle fermentations and immediately put on ice. One milliliter of the sample volume was then centrifuged (15,700*g*, 4 °C, 10 min, Centrifuge 5415 R, Eppendorf, Hamburg, Germany) and the supernatant stored at −20 °C prior to HPLC analysis. The cell growth was recorded as increase in OD_600_. The cell dry weight (CDW) was determined by filtering 1 mL of cell suspension through pre-weighed cellulose-acetate filter papers. After washing thoroughly with water, the filter paper was dried for 15 min in a microwave, cooled down in a desiccator, and weighed. Cell dry weights were recorded for YX, YG, and YGX fermentations and determined in triplicates. For analysis of colony forming units (CFU), the cell suspension was diluted with 0.9% (w/v) NaCl solution, and 1 mL of the appropriately diluted cell suspension was plated on YPD agar plates. Incubation was at 30 °C for 48 h. Extracellular fermentation products (ethanol, glycerol, xylitol, and acetic acid) and sugars (xylose and glucose) were analyzed by HPLC (Merck-Hitachi LaChrom system, L-7250 autosampler, L-7490 RI detector, L-7400 UV detector; Merck, Whitehouse Station, NJ). The system was equipped with an Aminex HPX-87H column and an Aminex Cation H guard column (both Bio-Rad, Hercules, CA). The operating temperature was 65 °C, and the flow rate of the mobile phase (5 mM sulfuric acid) was 0.6 mL/min.

#### Data processing and evaluations

The maximal specific growth rate (*µ*_max_; h^−1^) was determined as the slope of the linear region of the ln(OD_600_) vs time trajectory. Carbon balances were calculated with the assumption that 1 mol CO_2_ was formed per mol acetate and ethanol. For biomass yields, a C-molar weight of 26.4 g Cmol^−1^ was applied [34]. The specific uptake rates *q*_Glucose_ and *q*_Xylose_ were calculated by first plotting glucose and xylose concentrations against fermentation time. The resulting scatter plots were fitted with suitable equations, and the first derivatives of the fitted equations were used to calculate the volumetric uptake rates *Q* (g L^−1^ h^−1^). To calculate *q*_Glucose_ and *q*_Xylose_ (g g_CDW_^−1^ h^−1^), *Q* was further normalized to the CDW. Similar to previously published studies, both *q*_Glucose_ and *q*_Xylose_ decreased with reaction time. Thus, reported values herein represent arithmetic means of the first four determinations made within the initial phase of the reaction. Please note: In fermentations containing glucose *and* xylose (YGX, H-YG and H-YGX), both strains showed an initial phase where only glucose was consumed (“glucose phase”) and only subsequently xylose uptake started (“xylose phase”). *q*_Xylose_ therefore represents the arithmetic mean of the first four sampling points of the xylose phase. Based on the improved co-fermentation capacity of both evolved strains, however, it was not possible to separate the phases completely, resulting in residual glucose being present in the time frame when *q*_Xylose_ was determined.

### High-gravity SSCF

The SSCF fermentation strategy will be published in full detail in another publication [33], and will be only briefly summarized here. Seed cultures were prepared in shake flask cultures containing YPD medium. Subsequently, cell propagation was accomplished in batch followed by fed-batch cultivation in 3.6 L bioreactors (INFORS HT, Switzerland). The batch and the feed media contained molasses, hydrolyzate, and media supplements, and propagation was run at 35 °C under aerobic conditions. For the SSCF, the solid fraction of the pretreated wheat straw was utilized as substrate and the desired dry mass loading was adjusted with hydrolyzate to reduce water consumption. The SSCF was run in a multi-feed approach, feeding both the wheat straw solids and cells from the cell propagation reactor at predetermined time points [33, 35]. In total, 20% (w/w) water insoluble solids (WIS) were loaded to the reactor. The enzyme (Ctec2, Novozymes, Denmark) loading was 10 Filter Paper Units (FPU) per g WIS. Cells were added to maintain a CDW/WIS ratio of 0.02 g g^−1^. The SSCF was run at pH 5. A temperature profile was utilized, where the first 24 h were run at 35 °C after which the temperature was lowered to 30 °C. In total, the SSCF was run for 120 h. Samples were taken to measure external metabolites by HPLC, cell growth by total cell count, and cell viability by CFU [33].

## Results

### Shaken bottle fermentations

The strains IBB10B05 and KE6-12.A were compared in xylose and mixed glucose–xylose fermentations conducted in complex media or a hydrolyzate matrix. In this first part of the study, the fermentation performance of the strains was evaluated in anaerobic shaken bottle experiments. Yeast extract (10 g L^−1^) was the sole medium additive. As shown by us [36, 37] and others [38], yeast extract is sufficient for fermentations of pure sugar substrates as well as lignocellulosic hydrolyzates. It can replace mineral medium and expensive vitamin and trace element additives [36–38]. The hydrolyzate matrix represented the liquid fraction after dilute acid-catalyzed steam explosion, during which significant amounts of the hemicellulose were hydrolyzed into xylose (Additional file 1: Table S1). The hydrolyzate further contained inhibitory compounds including acetic acid, 5-hydroxymethylfurfural (HMF), and furfural (Additional file 1: Table S1). These experiments were, hence, designed to evaluate the robustness of the strains. Fermentations were either run at high cell density (starting OD_600_ ~5) or low cell density (starting OD_600_ ~0.1). High cell density was used to analyze the conversion capacity of the yeast strains. Because of the high starting OD_600_ and the limited nutrients in shaken bottle experiments, only marginal cell growth was observed and the OD_600_ doubled maximally once within the fermentation time. Variations in growth are reflected in the biomass yields (*Y*_Biomass_). To still be able to analyze the ability of the strains to grow anaerobically on the sugar substrates under the provided conditions, low cell density fermentations were additionally conducted.

#### Comparison of KE6-12.A and IBB10B05 in high cell density fermentations

**Fig. 1:**
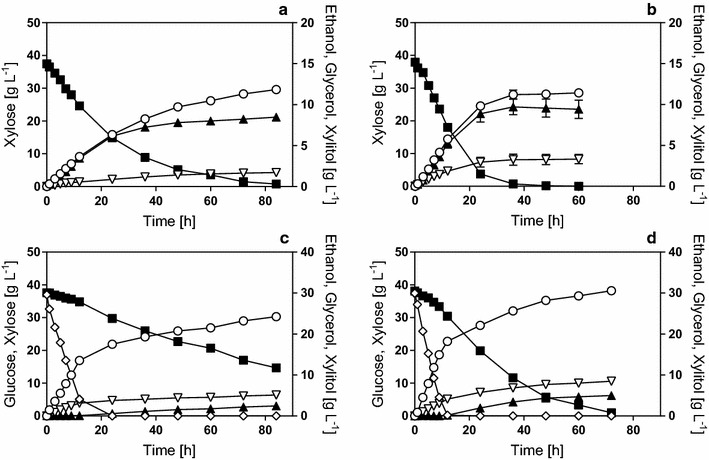
Time courses of shaken bottle fermentations in complex media supplemented with xylose or with glucose and xylose. Fermentations were performed in YX (**a**, **b**) and YGX (**c**, **d**) media using strains IBB10B05 (**a**, **c**) and KE6-12.A (**b**, **d**). The starting OD_600_ was 5. Data points are mean values from biological duplicates. *Error bars* indicate the spread. Symbols: Xylose (*filled squares*), glucose (*empty diamonds*), ethanol (*empty circles*), glycerol (*empty triangles*), and xylitol (*filled triangles*)

IBB10B05 and KE6-12.A were first analyzed in high cell density fermentations of xylose (YX) and glucose and xylose (YGX). The resulting time courses are depicted in Fig. 1. The physiological parameters calculated from the data are summarized in Table 1. In fermentations of xylose only, KE6-12.A was faster in metabolizing xylose than IBB10B05 (Fig. 1a, b), resulting in an almost twice as high *q*_Xylose_ (Table 1). The *Y*_Ethanol_ and *Y*_Xylitol_ were similar for both strains at ~0.30 and ~0.25 g g^−1^, respectively. In mixed glucose–xylose fermentations, KE6-12.A also showed faster sugar uptake (Fig. 1c, d) and the *q*_Glucose_ and *q*_Xylose_ were 1.3-fold and a 2.7-fold higher, respectively, than they were in IBB10B05. The *Y*_Ethanol_ was 0.40 g g^−1^ for both strains and the by-product distribution was also similar for the two strains (Table 1). In fermentations of the mixed sugar substrates, both strains showed some degree of true co-fermentation of glucose and xylose between 5 and 15 h fermentation time (Fig. 1).

**Table 1:** The physiological parameters of strains IBB10B05 and KE6-12.A in high cell density fermentations (starting OD_600_ 5) of xylose (YX) and glucose and xylose (YGX) in complex media

	YX		YGX^a^	
	IBB10B05	KE6-12A	IBB10B05	KE6-12A
*q*_Glucose_ [g g_CDW_^−1^ h^−1^]	–	–	0.92 ± 0.02	1.23 ± 0.03
*q*_Xylose_ [g g_CDW_^−1^ h^−1^]	0.34 ± 0.00	0.66 ± 0.04	0.10 ± 0.01	0.27 ± 0.02
*Y*_Ethanol_ [g g^−1^]	0.31 ± 0.00	0.30 ± 0.01	0.40 ± 0.00	0.40 ± 0.00
*Y*_Glycerol_ [g g^−1^]	0.04 ± 0.00	0.09 ± 0.01	0.09 ± 0.00	0.11 ± 0.00
*Y*_Xylitol_ [g g^−1^]	0.24 ± 0.00	0.25 ± 0.03	0.04 ± 0.00	0.07 ± 0.01
*Y*_Acetate_ [g g^−1^]	0.04 ± 0.00	0.01 ± 0.00	0.02 ± 0.00	0.01 ± 0.00
*Y*_Biomass_ [g g^−1^]	0.06 ± 0.01	0.04 ± 0.00	0.05 ± 0.00	0.02 ± 0.00
C-recovery [%]	100.8 ± 0.7	96.4 ± 2.3	97.5 ± 1.0	99.7 ± 0.2

Data represent the mean values and the spread between biological duplicates

^a^Yields are based on consumed xylose and glucose

**Fig. 2:**
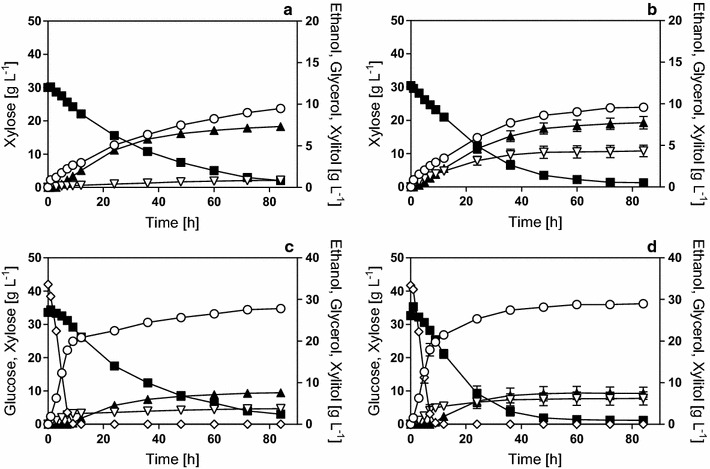
Time courses of shaken bottle fermentations of xylose or with glucose and xylose in a hydrolyzate matrix. Fermentations were performed in H-YX (**a**, **b**) and H-YGX (**c**, **d**) media using strains IBB10B05 (**a**, **c**) and KE6-12.A (**b**, **d**). The starting OD_600_ was 5. Data points are mean values from biological duplicates. *Error bars* indicate the spread. Symbols: Xylose (*filled squares*), glucose (*empty diamonds*), ethanol (*empty circles*), glycerol (*empty triangles*), and xylitol (*filled triangles*)

In the next step, strain performance was compared in an inhibitor-rich hydrolyzate. The hydrolyzate was supplemented with xylose (H-YX) and glucose and xylose (H-YGX). The time courses using IBB10B05 and KE6-12.A are depicted in Fig. 2. The corresponding physiological parameters are summarized in Table 2. For clarity reasons, glucose is not depicted in Fig. 2a and b, but the hydrolyzate contained a small amount of glucose (~2 g L^−1^, Additional file 1: Table S1), which was consumed by both strains at equal speed and was depleted within the first 2.5 h of fermentation. In fermentations of xylose, KE6-12.A showed a 1.4-fold higher *q*_Xylose_ than IBB10B05 did. The *Y*_Ethanol_ and *Y*_Xylitol_ were similar at ~0.31 and ~0.25 g g^−1^, respectively. The two strains, however, varied significantly in the formation of glycerol and acetate. KE6-12.A produced 0.15 g g^−1^ glycerol but no acetate. IBB10B05 produced 0.03 g g^−1^ glycerol but 0.04 g g^−1^ acetate. In fermentations of H-YGX, *q*_Xylose_ was 1.5-fold higher for KE6-12.A than for IBB10B05. *q*_Glucose_ (~2.1 g g_CDW_^−1^ h^−1^) and *Y*_Ethanol_ (0.39 g g^−1^) were similar for both strains. As in the fermentations of H-YX, KE6-12.A produced more glycerol and less acetate than IBB10B05, but the differences were smaller (Table 2). Glucose and xylose co-consumption was less pronounced for both strains in H-YGX as compared to YGX fermentations.

**Table 2:** The physiological parameters of strains IBB10B05 and KE6-12.A in high cell density fermentations (starting OD_600_ 5) of xylose (YX) and glucose and xylose (YGX) in a hydrolyzate matrix

	H-YX		H-YGX	
	IBB10B05	KE6-12.A	IBB10B05	KE6-12.A
*q*_Glucose_ [g g_CDW_^−1^ h^−1^]			2.10 ± 0.11	2.13 ± 0.22
*q*_Xylose_ [g g_CDW_^−1^ h^−1^]	0.30 ± 0.02	0.43 ± 0.03	0.24 ± 0.01	0.36 ± 0.03
*Y*_Ethanol_ [g g^−1^]	0.32 ± 0.02	0.31 ± 0.01	0.39 ± 0.00	0.40 ± 0.00
*Y*_Glycerol_ [g g^−1^]	0.03 ± 0.00	0.15 ± 0.03	0.05 ± 0.00	0.08 ± 0.01
*Y*_Xylitol_ [g g^−1^]	0.26 ± 0.01	0.24 ± 0.02	0.08 ± 0.00	0.08 ± 0.02
*Y*_Acetate_ [g g^−1^]	0.04 ± 0.00	0.00 ± 0.00	0.03 ± 0.00	0.01 ± 0.00
*Y*_Biomass_ [g g^−1^]	0.03 ± 0.00	0.03 ± 0.01	0.04 ± 0.00	0.03 ± 0.01
C-recovery [%]	100.1 ± 4.5	102.2 ± 1.9	96.7 ± 1.5	97.1 ± 0.5

Data represent the mean values and the spread between biological duplicates

Yields are based on consumed xylose and glucose

Addition of hydrolyzate affected the specific substrate uptake rates differently in the various experimental set-ups. In fermentations of xylose only (YX and H-YX, Tables 1, 2), the addition of hydrolyzate slowed down the xylose conversion in both strains, and *q*_Xylose_ was reduced 1.1- and 1.5-fold in IBB10B05 and KE6-12.A, respectively. When fermentations were conducted with mixed sugar substrates, addition of hydrolyzate instead enhanced *q*_Xylose_ as well as *q*_Glucose_ (Tables 1, 2). Thus, IBB10B05 showed a 2.3- and 2.4-fold increase in *q*_Glucose_ and *q*_Xylose_, respectively, in H-YGX as compared to YGX fermentations. In KE6-12.A, the difference was 1.7-fold (*q*_Glucose_) and 1.3-fold (*q*_Xylose_).

#### Comparison of KE6-12.A and IBB10B05 in low cell density fermentations

IBB10B05 and KE6-12.A were also compared in low cell density fermentations. The results are displayed in Table 3, which summarizes the maximal specific growth rate and the corresponding specific sugar uptake rates. The time courses and a summary of the metabolite yields of fermentations conducted without added hydrolyzate can be found in the Additional file 2: Figure S1 and Additional file 3: Table S2, respectively. Here, the two strains exhibited similar growth rates of 0.04 h^−1^ (YX) and 0.27 h^−1^ (YGX). In YX media, the *q*_Xylose_ was slightly higher for IBB10B05 than KE6-12.A (Table 3). In YGX media, both strains metabolized glucose at equal rate, but *q*_Xylose_ was 1.5-fold lower in IBB10B05 than in KE6-12.A (Table 3).

**Table 3:** Comparison of the maximal growth rates and specific substrate uptake rates of strains IBB10B05 and KE6-12A in low cell density fermentations (starting OD_600_ 0.1) in complex media and a hydrolyzate matrix containing xylose (YX and H-YX) or a combination of glucose and xylose (YGX and H-YGX)

	IBB10B05			KE6-12.A		
	*µ*_max_ [h^−1^]	*q*_Glucose_ [g g_CDW_^−1^ h^−1^]	*q*_Xylose_ [g g_CDW_^−1^ h^−1^]	*µ*_max_ [h^−1^]	*q*_Glucose_ [g g_CDW_^−1^ h^−1^]	*q*_Xylose_ [g g_CDW_^−1^ h^−1^]
YX	0.05 ± 0.00		0.77 ± 0.03	0.04 ± 0.00		0.68 ± 0.13
YGX	0.27 ± 0.01	1.35 ± 0.35	0.11 ± 0.02	0.27 ± 0.01	1.28 ± 0.20	0.17 ± 0.20
H-YX	0.13 ± 0.01		0.82 ± 0.06	0.17 ± 0.01		1.08 ± 0.04
H-YGX	0.21 ± 0.00	1.84 ± 0.22	0.52 ± 0.12	0.20 ± 0.01	1.58 ± 0.56	0.60 ± 0.03

Data represent the mean values and the spread between biological duplicates

The time courses of fermentations conducted in a hydrolyzate matrix are depicted in the Additional file 4: Figure S2 and the metabolic yields are summarized in Additional file 5: Table S3. Under these conditions the *µ*_max_ of both strains was similar at ~0.20 h^−1^ when mixed sugar substrates were used (H-YGX). In fermentations of xylose only (H-YX), IBB10B05 showed a 1.3-fold lower *µ*_max_ as compared to KE6-12.A. The specific glucose and xylose uptake rates in H-YGX fermentations varied only insignificantly, but IBB10B05 tended to convert glucose faster and xylose slower than KE6-12.A (Table 3). In H-YX fermentations, the *q*_Xylose_ of KE6-12.A was 1.3-fold higher as compared to IBB10B05.

In contrast to high cell density fermentations, addition of the hydrolyzate affected the specific sugar uptake rates positively in all experimental set-ups, irrespective of the sugar substrate or strain used (Table 3). It was further observed, that inoculation with low cell densities tended to result in higher specific sugar conversion rates than in fermentations started with large inocula (Tables 1, 2, and 3). This effect was stronger in IBB10B05, which showed an up to 2.7-fold higher *q*_Xylose_ in low cell density fermentations compared to the corresponding high cell density fermentation (Tables 1, 2, and 3).

### High-gravity multi-feed SSCF

**Fig. 3:**
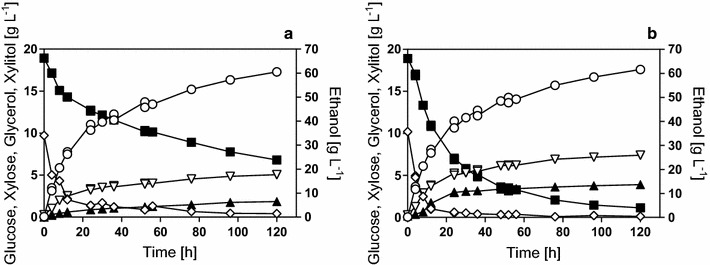
Time courses of mixed glucose–xylose fermentation in high-gravity SSCF fermentations with cell and substrate feed. Fermentations were performed using the strains B-Flow (flocculating IBB10B05; **a**) and KE-Flow (flocculating KE6-12.A; **b**). Solids were added after 0, 4, 12, 24, 36, and 52 h. Cells were fed after 0, 24, 36, and 52 h. Data of KE-Flow were taken from [33]. Symbols: Xylose (*filled squares*), glucose (*empty diamonds*), ethanol (*empty circles*), glycerol (*empty triangles*), and xylitol (*filled triangles*)

To compare the strains under more realistic process conditions, we conducted a high-gravity SSCF experiment with the flocculating variants of IBB10B05 (B-Flow) and KE6-12.A (KE-Flow). The process was operated with solids and cell feeding. This is a result of a series of development studies, which included the modeling and optimization of the cell and solids feeding strategy [35, 39], and the use of the flocculating yeast strains to simplify harvesting and handling of the yeast cells and potentially improving their inhibitor tolerance [33, 40]. The process has been designed for strain KE6-12.A with the aims of (a) maximizing the solids loading while controlling the apparent viscosity to reduce mass and heat transfer limitations, (b) controlling the amount of inhibitors added to the reactor, (c) keeping favorable glucose/xylose ratios to promote xylose fermentations, and (d) maintaining cell viability throughout the fermentations [35, 39]. The process was initially run at 35 °C to promote enzymatic hydrolysis. However, it has been clearly shown for KE-Flow (flocculating KE6-12.A) as well as for B-Flow (flocculating IBB10B05) that the combined stresses of the SSCF, i.e., high inhibitor and ethanol concentrations, have a much more severe impact on biomass growth at 35 °C than at 30 °C [33]. To accommodate both aspects of process efficiency, namely enzymatic hydrolysis rates and cell viability, the process was run at 35 °C for the first 24 h after which the temperature was lowered to 30 °C [33]. The resulting time courses of the SSCFs are depicted in Fig. 3. Table 4 shows the measured and calculated values for the xylose and the glucose uptake. Ethanol production and by-product formation are also summarized in the table. Both strains utilized almost all the available glucose monomers within the first 10 h of SSCF (Fig. 3). From that period on, the glucose concentration varied only slightly between 0.2 and 1.0 g L^−1^. This indicates that the cell viability, which was maintained above 40% for the major part of the fermentation (Additional file 6: Figure S3), was sufficient to continuously consume the glucose released by enzymatic hydrolysis. It further shows that the rate of the enzymatic hydrolysis and the rate of glucose consumption by the yeast cells were well matched. This validates the cell and solids feeding scheme not only for strain KE-Flow, for which it was developed, but also for B-Flow. Moreover, it demonstrates that the model-based feeding design [35, 39] is not strain specific, thus offering flexible application. In 120 h of fermentation, both strains produced approximately 60 g L^−1^ of ethanol. B-Flow consumed 12.2 g L^−1^ of the initially available xylose and produced only minor amounts of glycerol, xylitol, and acetate (Fig. 3; Table 4). KE-Flow converted almost all the initially available xylose (~19 g L^−1^) and produced slightly more glycerol and xylitol but less acetate than B-Flow.

**Table 4:** Sugar uptake and product formation in 120 h of SSCF fermentations using the flocculating strains B-Flow (IBB10B05) and KE-Flow (KE6-12.A)

	B-Flow	KE-Flow
Xylose consumption [g L^−1^]^a^	12.2	18.5
Glucose consumption [g L^−1^]^b^	136.5	139.3
Ethanol production [g L^−1^] (*Y*_Ethanol_ [g g^−1^])^c^	69.6 (0.47)	71.0 (0.45)
Glycerol production [g L^−1^] (*Y*_Glycerol_ [g g^−1^])^c^	5.2 (0.03)	7.8 (0.05)
Xylitol production [g L^−1^] (*Y*_Xylitol_ [g g^−1^])^c^	1.8 (0.01)	4.0 (0.03)
Acetate production [g L^−1^] (*Y*_Acetate_ [g g^−1^])^c^	1.7 (0.01)	0.1 (0.00)

^a^Consumed xylose under the assumption that no additional xylose was released by enzymatic hydrolysis

^b^Consumed glucose calculated based on the produced ethanol using the theoretical ethanol on glucose yield of 0.51 g g^−1^

^c^Metabolic yields based on the xylose and glucose consumption calculated as described in a and b

## Discussion

Laboratory evolution is an extremely powerful tool to enhance xylose-to-ethanol fermentation in yeasts. In this study, we compared two xylose-fermenting *S. cerevisiae* strains, IBB10B05 and KE6-12.A, which differ fundamentally in their metabolic and evolutionary history. IBB10B05 is based on the CEN.PK 113-5D genomic background and harbors an engineered NADH-preferring XR and a wild-type XDH [32, 41, 42]. It was evolved on mineral media with xylose as sole carbon source under strictly anaerobic conditions [20]. IBB10B05 was previously characterized in synthetic media [20, 37], in spent sulfite liquor [36] and in wheat straw hydrolyzates [37]. KE6-12.A harbors wild-type versions of *P. stipitis* XR and XDH and was evolved in a multitude of rounds, including chemostat evolution on xylose with increasing amounts of inhibitor-rich bagasse hydrolyzate under aerobic conditions ([25], Albers et al., unpublished). KE6-12.A was previously analyzed in fermentations of dilute lignocellulosic hydrolyzates [25] and high-gravity SSCFs [35, 39]. In this study, IBB10B05 and KE6-12.A were characterized and compared in identical experimental set-ups, with the aim of generating more information of how the strain background and the metabolic and the evolutionary engineering strategy affects the respective strain performance.

### KE6-12.A and IBB10B05 show similar *Y*_Ethanol_

**Fig. 4:**
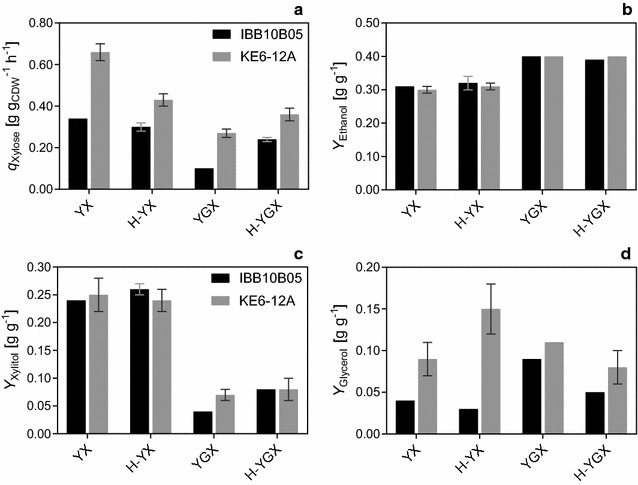
Comparison of xylose uptake rates and product yields of strains IBB10B05 and KEG-12.A in high cell density shaken bottle fermentations. Depicted are *q*_Xylose_ (**a**), *Y*_Ethanol_ (**b**), *Y*_Xyiltol_ (**c**), and *Y*_Glycerol_ (**d**) using strains IBB10B05 (*black bars*) and KE6-12.A (*gray bars*). Fermentations were conducted in complex media (YX and YGX) and a hydrolyzate matrix (H-YX and H-YGX) as indicated. Data were taken from Tables 1 and 2 and represent the mean values and the spread of biological duplicates

To facilitate comparison, the physiological parameters from high cell density shaken bottle fermentations are summarized in Fig. 4. Independent of the fermentation media, both strains showed approximately the same *Y*_Ethanol_, which was ~0.30 g g^−1^ in fermentations of xylose, and ~0.40 g g^−1^ in mixed glucose–xylose fermentations (Fig. 4b). In low cell density fermentations and SSCFs, the *Y*_Ethanol_ was also similar for both strains (Table 4; Additional file 3: Table S2, Additional file 5: Table S3). These results indicate that *Y*_Ethanol_ alone is not sufficient for detailed strain comparison. As described before, it is rather the biomass growth, the sugar consumption rates, the by-product formation, and the ethanol productivities that differ between various strains and which are sensitive to changes in the experimental set-up [6, 43, 44].

### Development of xylose uptake in KE6-12.A and IBB10B05

The *q*_Xylose_ of KE6-12.A exceeded that of IBB10B05 regardless of the fermentation medium used (Fig. 4a). KE6-12.A further showed a faster xylose conversion in low cell density fermentations (Table 3) and in SSCFs (Fig. 3). This is in accordance with evidence from previously published studies, that industrial strains are preferred progenitor strains to realize high substrate conversion rates [45–47]. However, in this study, the two strains do not only vary in their strain background. IBB10B05 and KE6-12.A also differ in their metabolic and evolutionary engineering strategy, individually designed to increase *q*_Xylose_.

Strain IBB10B05 incorporates XR, XDH, and XK enzymes with reported activities of ~1.2, ~0.9, and 1.9 U/mg_crude cell protein_, respectively [20]. These activities are significantly higher than corresponding activities reported for strain TMB3400 (XR ~ 0.08 U/mg_crude cell protein_, XDH ~ 0.22 U/mg_crude cell protein_, and XK ~ 0.08 U/mg_crude cell protein_ [21]), the parent strain of KE6-12.A. In accordance to flux control theory [48], accelerated xylose conversion in IBB10B05 was suggested to be mainly caused by high levels of XR, XDH, and XK activity [20, 49, 50]. One would expect therefore that IBB10B05 exhibit higher *q*_Xylose_ than KE6-12.A. TMB3400, in contrast, was shown to contain significantly enhanced levels of transporter proteins as result of evolution [21]. Furthermore, evolution for increased inhibitor resistance, as represented here by strain KE6-12.A, was associated with an increased expression of genes involved in the pentose phosphate pathway [51]. Increased flux through the pentose phosphate pathway could create a kinetic pull effect through the xylose assimilation pathway involving the XR, XDH, and XK catalyzed reactions. KE6-12.A further has a much longer laboratory evolution history than IBB10B05, including chemical mutagenesis, evolution for improved xylose conversion on pure sugar substrate [21], and chemostat experiments on xylose with inhibitor-rich bagasse hydrolyzate (Albers et al., unpublished). The last step alone already involved 102 generations, whereas IBB10B05 was evolved in just 61 generations in total [20].

We therefore speculate that the industrial strain background in combination with the prolonged laboratory evolution history, and the resulting traits elicited in KE6-12.A, overcompensated the effect of the high XR, XDH, and XK activities in IBB10B05.

### Impact of the lignocellulosic substrates on *q*_Xylose_

Lignocellulosic hydrolyzates contain inhibitors such as acetic acid, HMF, and furfural, all of which negatively impact cell viability, biomass growth, and ethanol productivity. The physiological parameter, which is most susceptible to inhibition in engineered *S. cerevisiae*, is *q*_Xylose_ [6, 43, 44]. In this study, the inhibitor tolerance of IBB10B05 and KE6-12.A was firstly evaluated by comparing the fermentation performance in shaken bottle fermentations with and without added hydrolyzate.

In high cell density fermentations of xylose only, addition of hydrolyzate reduced *q*_Xylose_ in both strains (YX and H-YX, Fig. 4a), but to different extents. In IBB10B05 this effect was much less pronounced than in KE6-12.A (Tables 1, 2). The likely reason for the strongly decreased *q*_Xylose_ in KE6-12.A is a loss of viability (measured in colony forming units, Additional file 7: Figure S4). In fermentations of H-YX, the cell viability decreased rapidly within the first 50 h to ~40% of the original value. In fermentation of YX only, no drop in viability was observed (Additional file 7: Figure S4). In contrast to KE6-12.A, the viability of IBB10B05 stayed equally constant at almost 100% over fermentation time, independent of the addition of hydrolyzate (Additional file 7: Figure S4).

However, it seems unlikely that the lignocellulose-derived inhibitors had a stronger inhibiting effect on KE6-12.A than they had on IBB10B05. Industrial strains were shown to be more inhibitor tolerant than laboratory strains [45–47], and KE6-12.A was evolved for increased inhibitor resistance ([25], Albers et al., unpublished). Moreover, KE6-12.A did not show a decrease in *q*_Xylose_ when hydrolyzate was added to low cell density fermentations of xylose (YX and H-YX, Table 3), which are more prone to inhibition by toxic compounds than are fermentations using large inocula [52].

It is likely that the observed differences are a result of the respective evolution strategy in combination with the experimental set-up used. In high cell density fermentations, which were designed to resemble larger scale applications, expensive media additives such as ergosterol or oleic acid were avoided. Both compounds are essential for anaerobic growth [53]. Thus, low cell density fermentations, designed to analyze differences in *µ*_max_, were supplemented with an ergosterol solution additionally containing Tween-80. The lack of these essential compounds in high cell density fermentations in combination with the strictly anaerobic conditions represents a significant stress on the yeast cell [53]. This stress was targeted by the evolution strategy of IBB10B05, which was kept anaerobic during the entire evolution procedure [20]. KE6-12.A, in contrast, was evolved under aerobic conditions [21, 25]. We would like to suggest, therefore, that the drop in both viability and *q*_Xylose_ of KE6-12.A was brought about by the lack of ergosterol and/or oleic acid under conditions of lignocellulose-derived stressors in the hydrolyzate.

In mixed glucose–xylose fermentation, addition of the hydrolyzate had a beneficial impact on the glucose and xylose uptake rates (Fig. 4; Tables 1, 2). This effect was even more pronounced in low cell density fermentations (Table 3; Additional file 2: Figure S1, Additional file 4: Figure S2). In these fermentations *q*_Xylose_ was affected positively by the hydrolyzate, even when just the fermentation of xylose was analyzed (H-YX and YX, Table 3). This “boosting” impact of the hydrolyzate can have several reasons. The low amounts of acetic acid and salts, which are present in the hydrolyzate (Additional file 1: Table S1), can exercise moderate stress on the yeast cells [54–56]. The resulting enhanced need for energy and redox equivalents can trigger an increase in the glycolytic flux, which in turn results in higher fermentation rates [56]. The hydrolyzate further contained small amounts of furfural and HMF (Additional file 1: Table S1), which can both act as electron acceptors, facilitating NADH re-oxidation [57, 58]. This also renders higher glycolytic rates possible. The increased glycolytic flux and the corresponding kinetic pull through the pathways upstream of glycolysis may have further positively affected the *q*_Xylose_ in IBB10B05 and KE6-12.A in fermentations with added hydrolyzate (Fig. 4a; Table 3).

The inhibitor tolerance of the two strains was further evaluated under the high severity conditions of the SSCF, where B-Flow (IBB10B05) and KE-Flow (KE6-12.A) showed a comparable glucose uptake, produced a similar amount of ethanol, and displayed comparable viability over fermentation time (Fig. 3; Additional file 6: Figure S3). This indicates that the two strains are equally tolerant against the high severity conditions, even though KE6-12.A, in contrast to IBB10B05, was evolved for increased inhibitor tolerance.

The inhibitor tolerance of yeast cells has been shown to depend on the overexpression of enzymes, which can reduce the lignocellulose-derived furaldehydes (e.g., HMF and furfural) into their less harmful corresponding alcohols [43, 58]. Responsible for the furaldehyde reductions are native enzymes, e.g., the alcohol dehydrogenase ADH6, and also the heterologous XR in engineered *S. cerevisiae* [43, 58, 59]. Overexpression of the XR has been further suggested to play a role in the stress response of xylose-fermenting *S. cerevisiae*, similar to native aldose reductases [18, 60]. Thus, the high XR activity in IBB10B05 might have increased the inhibitor tolerance to a similar extent in IBB10B05 as the metabolic alterations caused by evolutionary engineering did in KE6-12.A.

A common trait of both evolved yeast strains is the strongly accelerated xylose metabolism [20, 21, 25]. This increase in *q*_Xylose_ results in significantly improved ATP generation rates, which, in turn, not only increase the ethanol productivity, but also provide the means to cope with lignocellulose-derived stressors, e.g., organic acids [20, 43].

### Co-enzyme specificity of the XR and its impact on by-product formation

Another difference between IBB10B05 and KE6-12.A is the type of XR and XDH the strains have incorporated. Whereas IBB10B05 harbors an engineered NADH-preferring XR, which renders the xylose assimilation pathway redox neutral [32], KE6-12.A contains the wild-type enzymes. The mismatched co-enzyme usage of the latter is widely accepted to be the main reason for excessive xylitol formation [32, 41, 61, 62]. As summarized in Fig. 4c, however, no difference in xylitol yields was detected between the two strains. Instead, the main strain-dependent difference was found in the *Y*_Glycerol_ (Fig. 4d). Thus, in all high cell density shaken bottle fermentations (Fig. 4d), as well as in low cell density fermentations (Additional file 3: Table S2, Additional file 5: Table S3), and SSCFs (Table 4), KE6-12.A produced significantly more glycerol than IBB10B05. Glycerol, like xylitol, functions as a “redox-sink”; its formation serves to remove excess NADH [63]. It therefore seems likely that the comparably high glycerol formation in KE6-12.A is an indicator for redox imbalances caused by the unequal co-enzyme specificity of the XR and the XDH. This is supported by the fact that the largest difference between the *Y*_Glycerol_ of IBB10B05 and KE6-12.A was found in fermentations of xylose as sole sugar substrate (Fig. 4d).

Figure 4 further indicates that both strains showed an increased *Y*_Xylitol_ at high *q*_Xylose_ (Fig. 4a, c). This is in accordance with a previously published study on strain IBB10B05, in which *Y*_Xylitol_ was demonstrated to increase with the *q*_Xylose_ [36]. The underlying reason for this effect is probably a kinetic bottleneck at the level of the XDH [42, 49].

### *q*_Xylose_ in both strains is dependent on the glucose concentration

In all presented experiments, the *q*_Xylose_ was lower in mixed glucose–xylose fermentations (YGX, H-YGX) than in xylose fermentations only (YX, H-YX), irrespective of fermentation matrix, cell density, or strain used (Fig. 4a; Table 3). It is well known that glucose can inhibit *q*_Xylose_ in engineered *S. cerevisiae* (e.g., [64–66]), which natively does not harbor specific xylose transporter proteins [67, 68]. Although the homologous hexose transporters (e.g., Hxt1-7p) can facilitate xylose uptake, their affinity for glucose is so much higher that xylose uptake is inhibited even at high xylose-to-glucose ratios [67, 68]. In contrast, basal amounts of glucose (<2 g L^−1^, e.g., [66]) have been shown to positively affect *q*_Xylose_. Upregulation of transporter gene expression and an increase in glycolytic flux, which can create a kinetic pull through the xylose catabolism upstream of glycolysis, are likely the reasons for this [65–67]. The dependence of *q*_Xylose_ on the glucose concentration in engineered *S. cerevisiae* has been exemplified for the progenitor strain of IBB10B05, BP10001, which showed an increase of *q*_Xylose_ from 0.15 g g_CDW_^−1^ h^−1^ (no glucose) to 0.30 g g_CDW_^−1^ h^−1^ at glucose concentrations below 0.3 g L^−1^. At glucose concentrations above 1 g L^−1^, however, xylose uptake decreased rapidly and ceased completely at >5 g L^−1^ [66].

In low cell density fermentations conducted in this study, both strains exhibited a higher *q*_Xylose_ in H-YX than in YX media (Table 3). In line with previous evidence [36, 66], this was probably caused by the basal glucose concentration in the hydrolyzate (~2 g L^−1^, Additional file 1: Table S1), which stayed in the medium for ~12 h of low cell density fermentations and thus, likely positively affected *q*_Xylose_.

Figure 4a and Table 3 further indicate that inhibition by glucose on *q*_Xylose_ was stronger in complex media than in fermentations conducted in a hydrolyzate matrix. Interpretation of the effect is difficult. However, the result supports the notion that specific sugar conversion rates are complex manifestations of yeast physiology strongly dependent on the fermentation conditions.

Inhibition of xylose transport by glucose is also the reason for the sequential sugar uptake by engineered strains of *S. cerevisiae* [10, 11]. In this study, both strains showed a short phase of true sugar co-consumption in fermentations of complex media (Figs. 1, 2). In the SSCFs, the phase of glucose and xylose co-fermentation was even extended to the whole process, judging from the continued increase in ethanol and decrease in xylose concentrations (Fig. 3). The increased ability of evolved *S. cerevisiae* strains to co-consume glucose and xylose was described before [15, 16, 69] and has been ascribed to the overexpression of transporter proteins, xylose pathway enzymes, and enzymes of the pentose phosphate pathway [16, 20, 69]. This has been also demonstrated for the parent strain of KE6-12.A, TMB3400, and for IBB10B05 [20, 21].

The higher sugar co-consumption in SSCFs as compared to shaken bottle fermentations was probably a result of the presence of basal amounts of glucose (see “*q*_Xylose_ in both strains is dependent on the glucose concentration”), released by enzymatic hydrolysis, and the cell propagation strategy (see “Methods”). Continuous cultivation of yeast on inhibitor-rich medium containing high amounts of xylose can promote the xylose fermentation capacity, inhibitor tolerance, and sugar co-consumption by short-term adaptation [70, 71].

## Conclusion

In this study, key physiological parameters of KE6-12.A and IBB10B05 were compared to evaluate the influence of the metabolic and evolutionary engineering strategies on strain performance. Despite minor differences in the physiological characteristics of the two strains, the global fermentation performance was remarkably convergent. These results indicate that the individual specific traits of the two strains, which were elicited by the respective metabolic or evolutionary engineering strategies, affected the physiological parameters in different ways and to varying extents. They furthermore highlight the importance of comparative strain evaluation across laboratories to dissect the benefits of individual specific traits brought about by strain engineering on the global fermentation performance.

## Additional files

**Additional file 1: Table S1.** Composition of the solid and the liquid fraction (here denoted hydrolyzate) of the pretreated wheat straw. Data were taken from [33].

**Additional file 2: Figure S1.** Time courses of low cell density shaken bottle fermentations in complex media supplemented with xylose or with glucose and xylose. Fermentations were performed in YX (a, b) and YGX (c, d) media using strains IBB10B05 (a, c) and KE6-12.A (b, d). The starting OD_600_ was 0.1. Data points are mean values from biological replicates. Error bars indicate the spread. Symbols: Xylose (filled squares), glucose (empty diamonds), ethanol (empty circles), glycerol (empty triangles), xylitol (filled triangles), and OD_600_ (crosses and dashed lines).

**Additional file 3: Table S2.** Comparison of the physiological parameters of strains IBB10B05 and KE6-12.A in low cell density fermentations (starting OD_600_ 0.1) of xylose (YX) and glucose and xylose (YGX).

**Additional file 4: Figure S2.** Time courses of low cell density shaken bottle fermentations with xylose or with glucose and xylose in a hydrolyzate matrix. Fermentations were performed in H-YX (a, b) and H-YGX (c, d) media using strains IBB10B05 (a, c) and KE6-12.A (b, d). The starting OD_600_ was 0.1. Data points are mean values from biological replicates. Error bars indicate the spread. Symbols: Xylose (filled squares), glucose (empty diamonds), ethanol (empty circles), glycerol (empty triangles), xylitol (filled triangles), and OD_600_ (crosses and dashed lines).

**Additional file 5: Table S3.** Comparison of the physiological parameters of strains IBB10B05 and KE6-12.A in low cell density fermentations (starting OD_600_ 0.1) of xylose (H-YX) and mixed glucose-xylose (H-YGX) in a hydrolyzate matrix.

**Additional file 6: Figure S3.** Comparison of the change in viability over time in high gravity SSCF fermentations. Depicted are the colony forming units (CFU) per total cell count using B-Flow (IBB10B05; panel a) and KE-Flow (KE6-12A; panel b). The starting OD_600_ was 5. Data represent mean values of 3 counted plates. Data for KE-Flow were taken from [33]. Error bars indicate the spread.

**Additional file 7: Figure S4.** The change in viability over time in shaken bottle fermentations. Depicted are the colony forming units (CFU) per OD_600_ value, relative to the value at t = 0 h. Fermentations were conducted in complex media (a) and a hydrolyzate matrix (b) supplemented with xylose (circles) or glucose and xylose (triangles) using strains IBB10B05 (filled symbols) and KE6-12.A (empty symbols). The starting OD_600_ was 5. Data points are mean values from biological replicates. Error bars indicate the spread.
