# Supplementary material for: Saccharomyces cerevisiae strain comparison in glucose–xylose fermentations on defined substrates and in high-gravity SSCF: convergence in strain performance despite differences in genetic and evolutionary engineering history
Source: Biotechnol Biofuels. 2017 Sep 4;10:205. doi: 10.1186/s13068-017-0887-9 (PMC5584037; doi:10.1186/s13068-017-0887-9)
Supplement: Supplementary file 1 — Additional file 1: Table S1. Composition of the solid and the liquid fraction (here denoted hydrolyzate) of the pretreated wheat straw. Data were taken from [33]. [file 13068_2017_887_MOESM1_ESM.docx]

| Solid fraction | Components in [%] WIS |
| --- | --- |
| Glucan | 42.4 |
| Xylan | 2.6 |
| Mannan | 0.2 |
| Galactan | *n.d.* |
| Arabinan | 0.1 |
| Lignin | 41.7 |
| Total | 87.0 |
| Liquid fraction | Components in [g/L] |
| Glucose | 2.6 |
| Xylose | 22.7 |
| Mannose | 0.5 |
| Galactose | 1.0 |
| Arabinose | 2.8 |
| Acetic acid | 3.2 |
| Furfural | 0.8 |
| HMF | 0.4 |

*n.d. – not detectable*
