# Supplementary figures and images for: Saccharomyces cerevisiae strain comparison in glucose–xylose fermentations on defined substrates and in high-gravity SSCF: convergence in strain performance despite differences in genetic and evolutionary engineering history
Source: Biotechnol Biofuels. 2017 Sep 4;10:205. doi: 10.1186/s13068-017-0887-9 (PMC5584037; doi:10.1186/s13068-017-0887-9)

**a**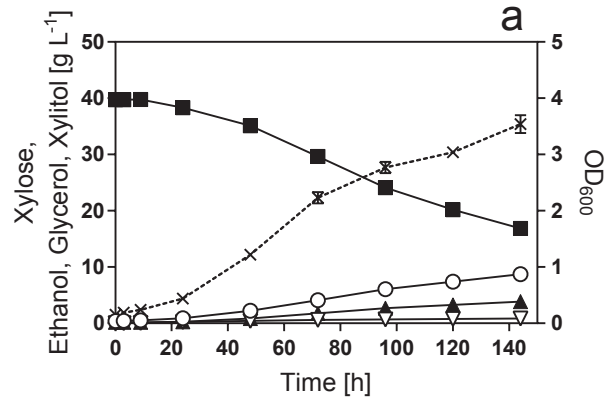**b**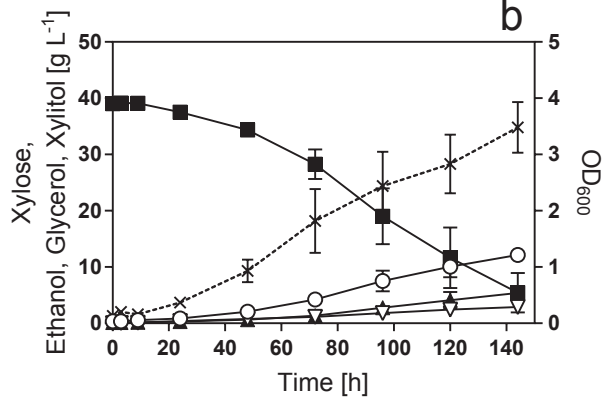**c**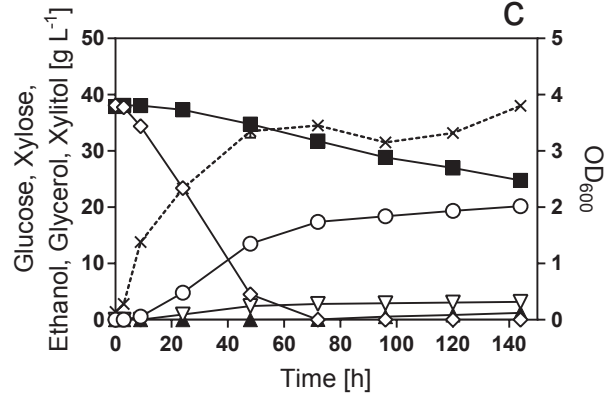**d**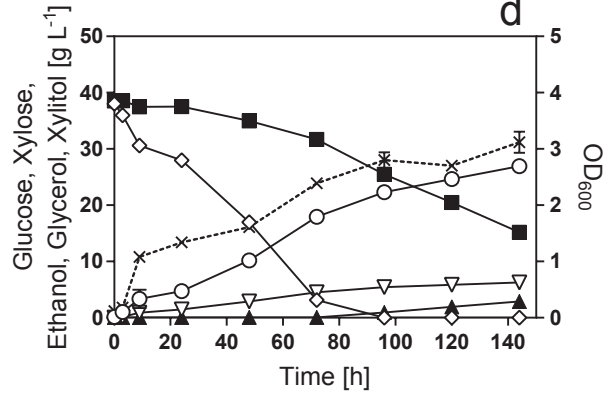

Supplement: Supplementary file 2 — Additional file 2: Figure S1. Time courses of low cell density shaken bottle fermentations in complex media supplemented with xylose or with glucose and xylose. Fermentations were performed in YX (a, b) and YGX (c, d) media using strains IBB10B05 (a, c) and KE6-12.A (b, d). The starting OD600 was 0.1. Data points are mean values from biological replicates. Error bars indicate the spread. Symbols: Xylose (filled squares), glucose (empty diamonds), ethanol (empty circles), glycerol (empty triangles), xylitol (filled triangles), and OD600 (crosses and dashed lines). [file 13068_2017_887_MOESM2_ESM.pdf]

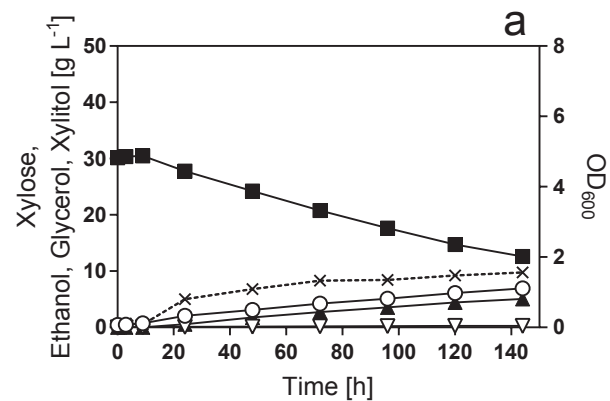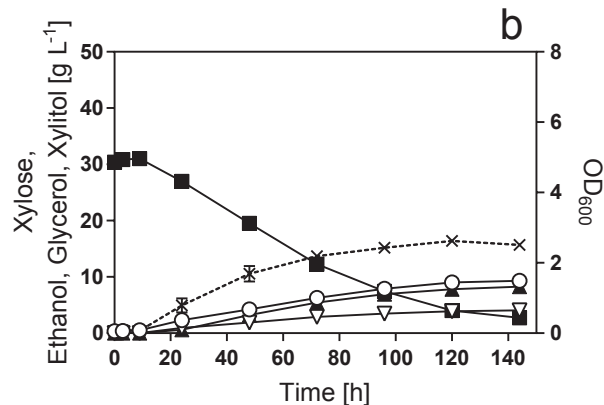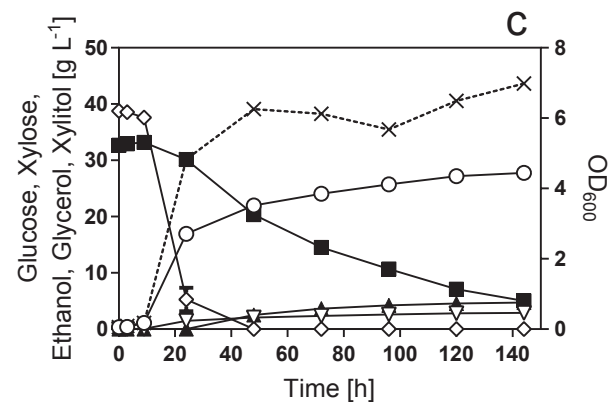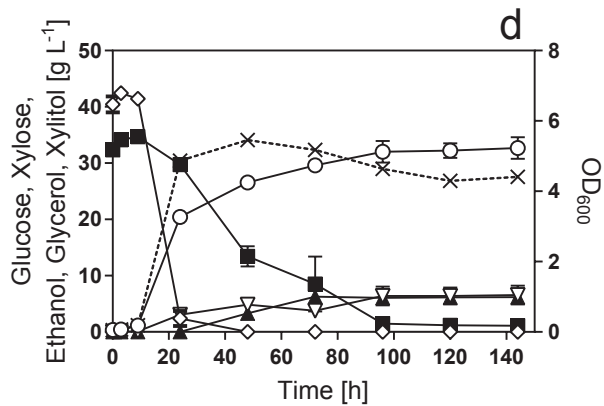

Supplement: Supplementary file 4 — Additional file 4: Figure S2. Time courses of low cell density shaken bottle fermentations with xylose or with glucose and xylose in a hydrolyzate matrix. Fermentations were performed in H-YX (a, b) and H-YGX (c, d) media using strains IBB10B05 (a, c) and KE6-12.A (b, d). The starting OD600 was 0.1. Data points are mean values from biological replicates. Error bars indicate the spread. Symbols: Xylose (filled squares), glucose (empty diamonds), ethanol (empty circles), glycerol (empty triangles), xylitol (filled triangles), and OD600 (crosses and dashed lines). [file 13068_2017_887_MOESM4_ESM.pdf]

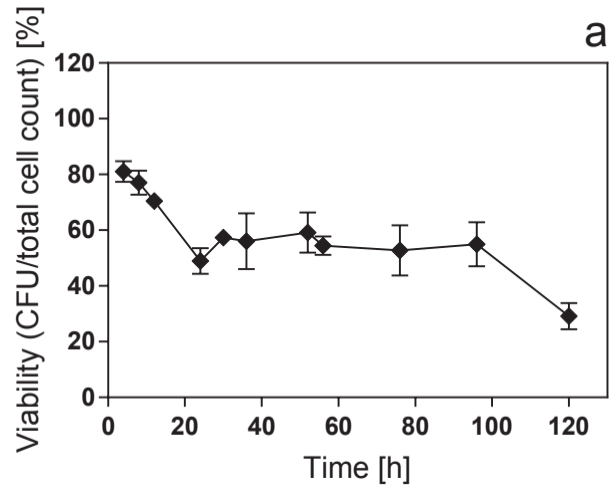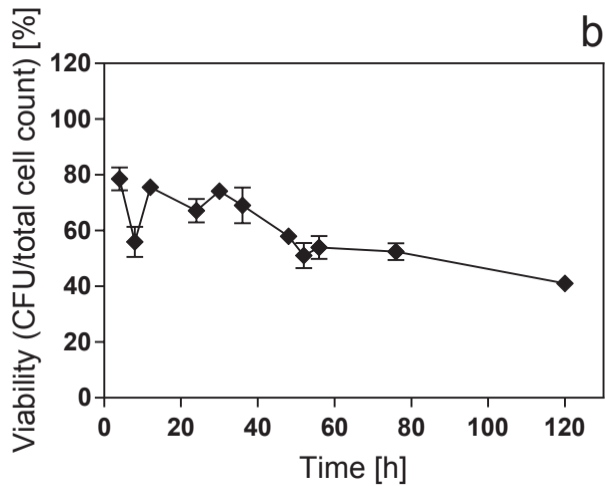

Supplement: Supplementary file 6 — Additional file 6: Figure S3. Comparison of the change in viability over time in high gravity SSCF fermentations. Depicted are the colony forming units (CFU) per total cell count using B-Flow (IBB10B05; panel a) and KE-Flow (KE6-12A; panel b). The starting OD600 was 5. Data represent mean values of 3 counted plates. Data for KE-Flow were taken from [33]. Error bars indicate the spread. [file 13068_2017_887_MOESM6_ESM.pdf]

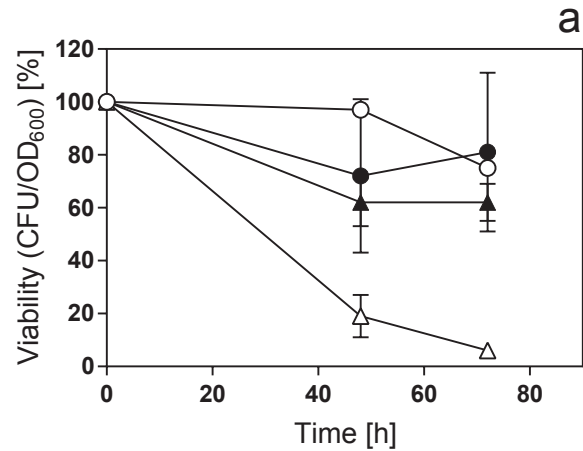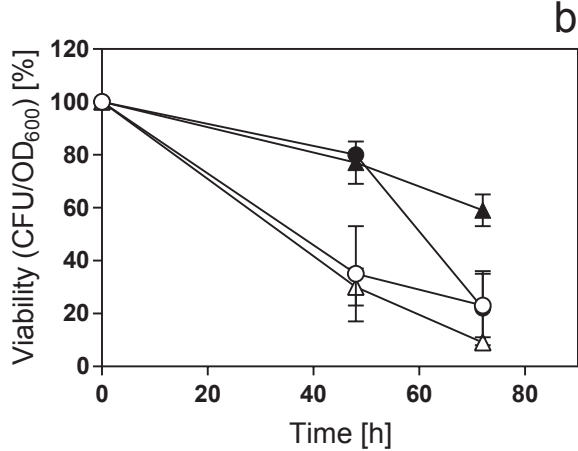

Supplement: Supplementary file 7 — Additional file 7: Figure S4. The change in viability over time in shaken bottle fermentations. Depicted are the colony forming units (CFU) per OD600 value, relative to the value at t = 0 h. Fermentations were conducted in complex media (a) and a hydrolyzate matrix (b) supplemented with xylose (circles) or glucose and xylose (triangles) using strains IBB10B05 (filled symbols) and KE6-12.A (empty symbols). The starting OD600 was 5. Data points are mean values from biological replicates. Error bars indicate the spread. [file 13068_2017_887_MOESM7_ESM.pdf]
