# Supplementary material for: Saccharomyces cerevisiae strain comparison in glucose–xylose fermentations on defined substrates and in high-gravity SSCF: convergence in strain performance despite differences in genetic and evolutionary engineering history
Source: Biotechnol Biofuels. 2017 Sep 4;10:205. doi: 10.1186/s13068-017-0887-9 (PMC5584037; doi:10.1186/s13068-017-0887-9)
Supplement: Supplementary file 3 — Additional file 3: Table S2. Comparison of the physiological parameters of strains IBB10B05 and KE6-12.A in low cell density fermentations (starting OD600 0.1) of xylose (YX) and glucose and xylose (YGX). [file 13068_2017_887_MOESM3_ESM.docx]

|  | YX | |  | YGX | |
| --- | --- | --- | --- | --- | --- |
|  | IBB10B05 | KE6-12.A |  | IBB10B05 | KE6-12.A |
| *Y*_Ethanol_ [g/g] | 0.36 | 0.36 |  | 0.42 | 0.43 |
| *Y*_Glycerol_ [g/g] | 0.04 | 0.09 |  | 0.07 | 0.11 |
| *Y*_Xylitol_ [g/g] | 0.16 | 0.14 |  | 0.03 | 0.03 |
| *Y*_Acetate_ [g/g] | 0.02 | 0.01 |  | 0.00 | 0.01 |
| *Y*_Biomass_ [g/g] | 0.07 | 0.05 |  | 0.03 | 0.02 |
| C-recovery [%] | 101.7 | 96.0 |  | 94.4 | 101.4 |
