# Supplementary material for: Saccharomyces cerevisiae strain comparison in glucose–xylose fermentations on defined substrates and in high-gravity SSCF: convergence in strain performance despite differences in genetic and evolutionary engineering history
Source: Biotechnol Biofuels. 2017 Sep 4;10:205. doi: 10.1186/s13068-017-0887-9 (PMC5584037; doi:10.1186/s13068-017-0887-9)
Supplement: Supplementary file 5 — Additional file 5: Table S3. Comparison of the physiological parameters of strains IBB10B05 and KE6-12.A in low cell density fermentations (starting OD600 0.1) of xylose (H-YX) and mixed glucose-xylose (H-YGX) in a hydrolyzate matrix. [file 13068_2017_887_MOESM5_ESM.docx]

|  | H-YX | |  | H-YGX | |
| --- | --- | --- | --- | --- | --- |
|  | IBB10B05 | KE6-12.A |  | IBB10B05 | KE6-12.A |
| *Y*_Ethanol_ [g/g] | 0.32 | 0.30 |  | 0.42 | 0.42 |
| *Y*_Glycerol_ [g/g] | 0.01 | 0.14 |  | 0.04 | 0.08 |
| *Y*_Xylitol_ [g/g] | 0.24 | 0.27 |  | 0.06 | 0.06 |
| *Y*_Acetate_ [g/g] | 0.03 | 0.00 |  | 0.02 | 0.00 |
| *Y*_Biomass_ [g/g] | 0.03 | 0.04 |  | 0.04 | 0.04 |
| C-recovery [%] | 93.7 | 100.9 |  | 98.3 | 99.0 |
